# Spatial Variation in Stream-Water Bacterial Communities Across Sites Differing in Wild Chinese Giant Salamander Detection Frequency

**DOI:** 10.3390/biology15181647

**Published:** 2026-09-18

**Authors:** Xinyao Yang, Junyi Chen, Shiqiang Chen, Song Liu, Da Qiu, Zhangnian Qin, Xingmin Tuo, Lirong Chen, Lei He

**Affiliations:** 1 School of Intelligent Science and Engineering, Hubei Minzu University, Enshi 445000, China; 202430323@hbmzu.edu.cn (X.Y.);; 2 Key Laboratory of Green Manufacturing of Super-Light Elastomer Materials, State Ethnic Affairs Commission, Hubei Minzu University, Enshi 445000, China; 3 Hubei Xianfeng Zhongjian River Giant Salamander National Nature Reserve Management Center, Enshi 445000, China

**Keywords:** Chinese giant salamander, environmental microbiome, wildlife microbiology, stream bacterial community, conservation monitoring

## Abstract

Wildlife monitoring and environmental microbiome profiling provide complementary perspectives for assessing habitats of protected aquatic species. This study integrated long-term computer vision monitoring, 16S rRNA amplicon sequencing, and water-quality measurements to examine stream sites differing in the camera-confirmed detection frequency of wild Chinese giant salamanders (*Andrias davidianus*). Monitoring from July 2024 to January 2026 recorded 47 independent detection events, of which 35 occurred at S1. S1 was therefore designated as the high-frequency detection site, while S2–S6 served as comparison sites. Stream-water bacterial communities were characterized from one composite sample per site, together with measurements of water temperature, pH, dissolved oxygen, conductivity, ammonia nitrogen, and nitrate nitrogen. S1 differed from several comparison sites in bacterial taxonomic composition, alpha-diversity estimates, and site-specific ASVs. Comamonadaceae, *Rhodoferax*, and *Flavobacterium* showed relatively high abundances at S1. However, RDA, envfit, Mantel, partial RDA, and variation-partitioning analyses found no significant independent relationship between the measured water-quality variables and bacterial-community variation. The observed spatial patterns likely reflect interactions among water quality, hydrology, substrate, riparian shading, refuge availability, organic-matter inputs, and anthropogenic disturbance. These results establish an exploratory spatial baseline and support the use of stream-water bacterial communities as a complementary component of Chinese giant salamander habitat monitoring.

## Simple Summary

The Chinese giant salamander is a protected aquatic species that depends on high-quality mountain-stream habitats. This study combined long-term camera monitoring, water-quality measurements, and stream-water bacterial-community analysis at six sites in a national nature reserve. Site S1 had the highest frequency of confirmed Chinese giant salamander detections and showed distinct bacterial-community characteristics, including relatively high abundances of Comamonadaceae, Rhodoferax, and Flavobacterium. The results indicate that camera monitoring and environmental microbiome data can provide complementary information on habitat conditions. Integrating biological detections, water-quality indicators, hydrological features, substrate conditions, riparian vegetation, and microbial-community characteristics can support more comprehensive monitoring and conservation management for the Chinese giant salamander.

## 1. Introduction

The continuing loss of global biodiversity has become a major ecological challenge, and the conservation of endangered species and restoration of their habitats are essential for maintaining ecosystem structure, function, and stability [1,2,3]. The Chinese giant salamander (*Andrias davidianus*) is a large freshwater amphibian and an indicator species of considerable conservation importance in mountain-stream ecosystems [4,5,6]. Wild populations continue to face substantial pressures resulting from habitat fragmentation, water pollution, human disturbance, and difficulties in population restoration, making the protection of native stream habitats an important component of regional biodiversity conservation [7,8]. Chinese giant salamanders are sensitive to water temperature, dissolved oxygen, water quality, shelter availability, and anthropogenic disturbance and generally reside in clean, oxygen-rich mountain streams containing abundant rock crevices and relatively low levels of disturbance [9,10]. Identifying their high-frequency detection sites from the perspective of aquatic environmental and microhabitat characteristics may therefore support refined reserve management and habitat assessment.

From an integrated host–microbiome–environment perspective, aquatic wildlife is continuously exposed to both host-associated microorganisms inhabiting the skin, oral cavity, stomach, and intestine and environmental microbial communities occurring in the surrounding water, sediment, and soil. Stream bacterial communities are shaped by physicochemical conditions, hydrological processes, nutrient inputs, substrate characteristics, and other local environmental factors and can therefore provide complementary information on spatial differences among aquatic microhabitats [11].

Experimental studies indicate that environmental microbial exposure may influence some components of the host-associated microbiota, although the magnitude of this influence differs among anatomical niches. In captive-bred Chinese giant salamander larvae, exposure to habitat river sediment altered oral bacterial community composition and multi-organ gene expression, whereas the skin, stomach, and intestinal microbiota were comparatively more stable [12]. The gastrointestinal microbiota of Chinese giant salamanders also varies with host age and developmental stage [13], changes during growth [14], and may be modified following reintroduction into the natural environment [15]. These findings indicate that environmental exposure and host internal factors jointly contribute to the formation of host-associated microbial communities.

Evidence from other animals further supports interactions among habitat conditions, diet, and gastrointestinal microbiota. In farmland frogs, habitat-associated dietary differences were accompanied by changes in dominant intestinal microorganisms [16]. Seasonal interactions between diet and gut microbiota have also been associated with environmental adaptation in high-altitude yaks [17]. Although results from mammals cannot be directly extrapolated to amphibians, they provide broader evidence that host diet, environmental conditions, and gastrointestinal microbiota can change together.

Host-associated microorganisms may also contribute to amphibian health and environmental adaptation through their relationships with pathogens. Experimental introduction of protective cutaneous bacteria reduced fungal infection in amphibian hosts [18], while variation in frog skin bacterial diversity has been associated with host susceptibility to *Batrachochytrium dendrobatidis* [19]. Amphibian skin microbial communities can vary over time, even when their predicted pathogen-inhibitory functions remain comparatively stable [20]. Environmental fluctuations may further alter interactions among amphibian hosts, skin-associated bacteria, and fungal pathogens, thereby changing relative survival outcomes under different environmental conditions [21]. Collectively, these studies support a biologically plausible connection among environmental microbial exposure, host-associated microbiota, host physiology, and environmental adaptation.

Nevertheless, Chinese giant salamander occurrence and activity are multifactorial and cannot be attributed to environmental microorganisms alone. Water temperature, water quality, hydrological conditions, habitat structure, food resources, host age, health status, and other biological and environmental variables may act simultaneously. Illumination may also influence developmental, physiological, biochemical, and behavioural processes in fish and amphibians [22]. Moreover, effects mediated through microbial colonization, immune responses, nutrient utilization, or metabolic regulation may occur after a temporal delay. Testing such delayed pathways requires repeated and synchronized measurements of environmental microbiomes, host-associated microbiomes, environmental conditions, physiology, and animal activity. Spatial correspondence between Chinese giant salamander detection frequency and environmental bacterial communities alone cannot demonstrate that environmental microorganisms cause changes in Chinese giant salamander behaviour.

High-throughput sequencing technologies have been widely applied to aquatic microbial ecology. Stream bacterial communities may respond to physicochemical conditions and local environmental filtering [23], and 16S rRNA amplicon sequencing can characterize bacterial taxonomic composition, relative abundance, diversity, and spatial variation. However, taxonomic profiles derived from 16S rRNA sequencing cannot independently demonstrate microbial metabolic activity, ecological function, host colonization, or physiological effects [24]. Standardized bioinformatic workflows can improve the reproducibility of bacterial-community characterization and ASV inference [25,26], while freshwater bacterial data can provide descriptive information on environmental and land-use differences among stream sites [27].

It should be emphasized that the molecular analysis conducted in this study targeted bacterial community DNA in stream-water samples rather than species-specific environmental DNA or host-associated microbiota of the Chinese giant salamander. The environmental bacterial data therefore did not represent Chinese giant salamander oral, skin, gastric, or intestinal microbiomes. Stream-water bacterial communities were treated as descriptive indicators of local microecological conditions rather than as direct evidence of gastrointestinal colonization, disease resistance, metabolic regulation, or behavioural effects.

Advances in artificial intelligence and computer vision have increasingly enabled long-term and low-disturbance wildlife monitoring. Camera-based monitoring and automated recognition can continuously screen large volumes of field video and provide information on the spatial distribution and frequency of confirmed wildlife detections [28,29,30,31,32,33,34]. This approach is particularly useful for Chinese giant salamanders because wild individuals generally occur at low densities, exhibit cryptic habits, and are unsuitable for repeated capture. Nevertheless, visual detection can be affected by camera placement, field of view, illumination, water transparency, target occlusion, equipment operation, animal movement pathways, and model detection probability. Consequently, sites with relatively frequent camera-confirmed detections should not be equated directly with greater individual abundance, higher locomotor activity, or core habitat status.

Against this background, this study developed a site-scale analytical framework integrating long-term computer vision monitoring, stream-water bacterial-community analysis, and aquatic environmental measurements. First, automated model screening followed by manual verification was used to identify stream sites differing in the frequency of confirmed wild Chinese giant salamander detections. Second, 16S rRNA amplicon sequencing was used to characterize the taxonomic composition, diversity, and structure of stream-water bacterial communities at six sampling sites. Third, physicochemical measurements collected during microbiome sampling and long-term environmental sensor records were used to explore associations between measured environmental conditions and bacterial-community variation. S1 had the highest frequency of confirmed detections during the monitoring period and was therefore designated as the high-frequency detection site. This designation describes relative detection frequency under the present monitoring configuration and does not define S1 as a core habitat.

In the present study, camera-confirmed detection records and independent detection events were used to describe site-level Chinese giant salamander detection frequency. These measures do not represent individual-level behavioural intensity, movement speed, physiological activity, individual abundance, or population density. This study did not conduct individual behavioural experiments or collect Chinese giant salamander oral, skin, gastric, or intestinal microbiome samples. Because only one high-frequency detection site was included and adjacent water subsamples from each site were pooled before sequencing, the analysis focused on spatial correspondence among confirmed Chinese giant salamander detections, stream-water bacterial communities, and measured aquatic conditions. It was not designed to determine whether environmental microorganisms influenced Chinese giant salamander behaviour, whether the presence of the species altered stream-water bacterial communities, or whether stream-water microorganisms colonized the host gastrointestinal tract.

## 2. Materials and Methods

### 2.1. Study Area and Sampling Sites

**Figure 1:**
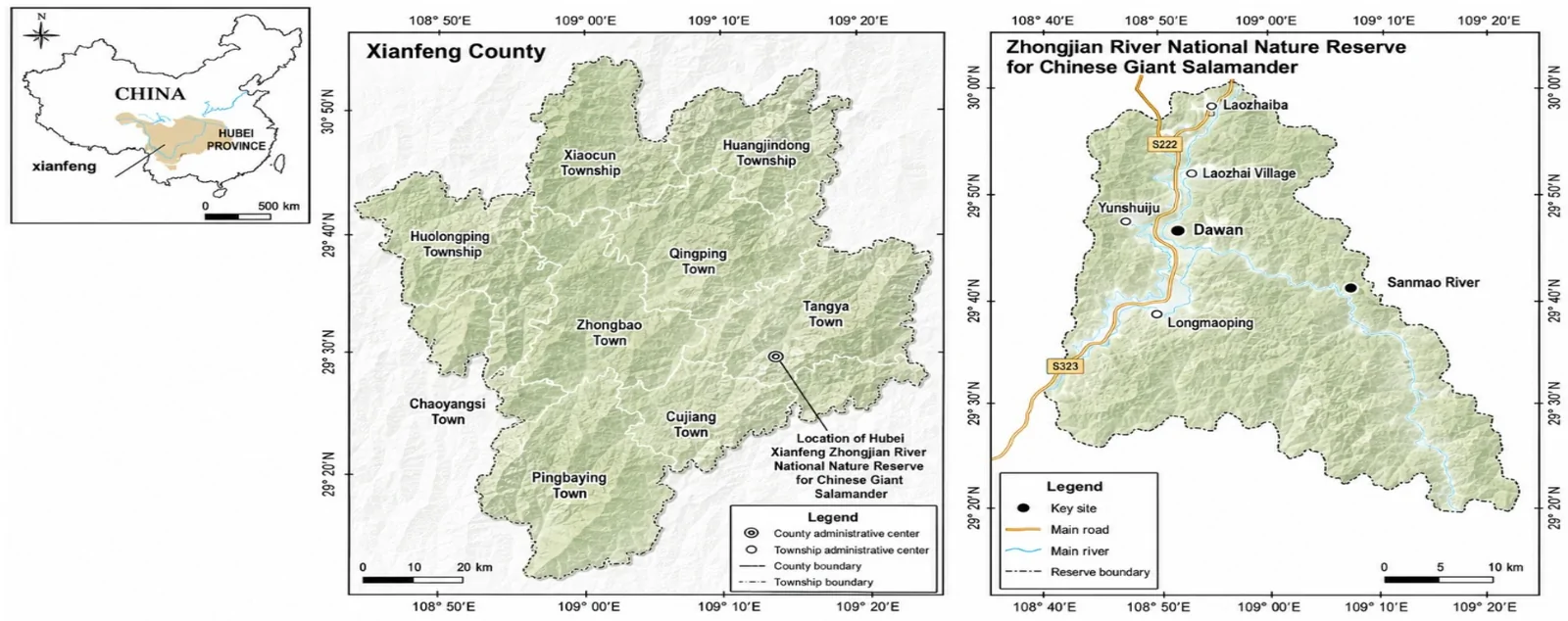
Geographic location of the Zhongjian River Giant Salamander National Nature Reserve in Xianfeng, Hubei.

The Hubei Xianfeng Zhongjian River Giant Salamander National Nature Reserve is located in the Wuling Mountains and has a core area of 359.5 ha. The reserve was established to protect the Chinese giant salamander (*Andrias davidianus*) and its natural habitat [31,33]. It is characterized by karst mountain streams, relatively clear water, and forest coverage exceeding 80%. Based on computer vision monitoring, this study selected S1 as the high-frequency detection site and S2–S6 as comparison sites. A total of six monitoring and sampling sites were therefore included; see Figure 1.

### 2.2. Computer Vision Monitoring System and Definition of the High-Frequency Detection Site

To document camera-confirmed detections of wild Chinese giant salamanders in streams within the reserve, computer vision monitoring equipment was deployed near sites S1–S6. The monitoring system consisted of surveillance cameras, solar-power modules, auxiliary illumination modules, edge-computing units, communication modules, and waterproof mounting structures. Field videos were captured using TL-IPC642-A cameras (TP-Link Technology Co., Ltd., Shenzhen, China). Camera locations were selected with reference to reserve patrol records, historical detections, stream-channel structure, and the distribution of caves and rock crevices. The camera fields of view covered the main channel, cave entrances, and concealed stream sections where Chinese giant salamanders were likely to pass or remain.

The monitoring system operated from July 2024 to January 2026. Captured video frames were initially screened using the YOLOv11-EWL Chinese giant salamander object-detection model developed by our research team. On an independent test set, the model achieved a recall of 94.85%, precision of 95.39%, F1 score of 95.12%, and inference speed of 77.20 frames s^−1^, with a storage size of 11.56 MB. Automated model outputs were used only for preliminary screening and were not treated as final confirmed detection records.

**Figure 2:**
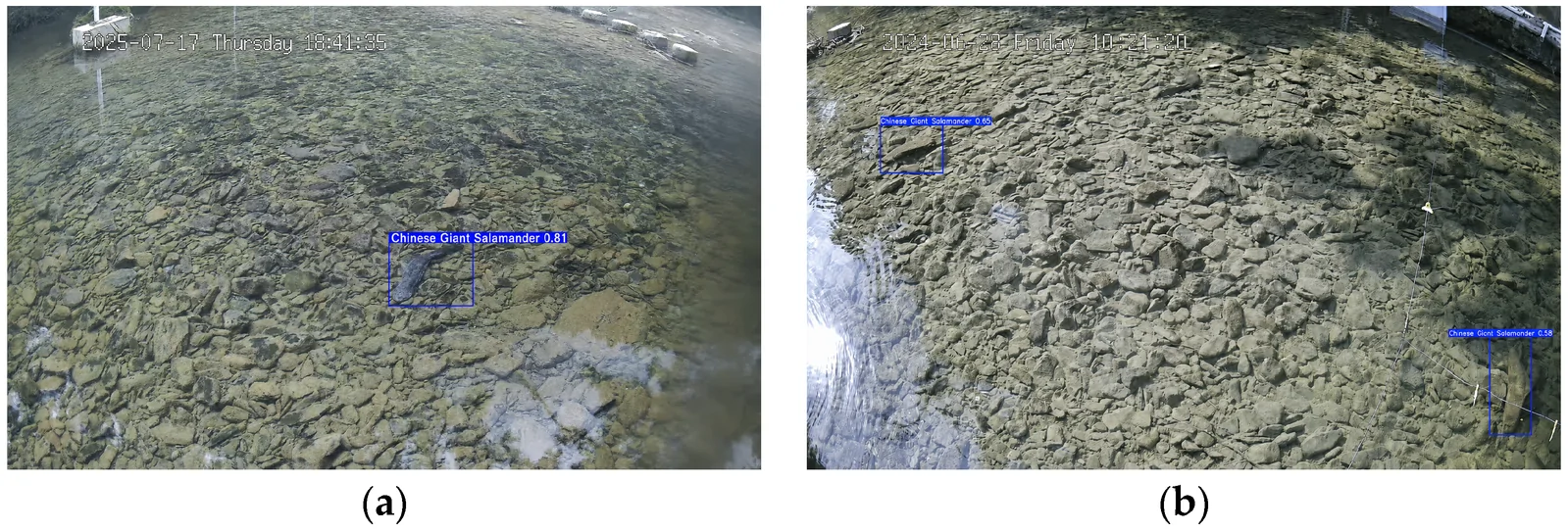
Test results of the Chinese giant salamander detection model. (**a**) A correctly detected Chinese giant salamander confirmed by manual review; and (**b**) a false-positive detection excluded during manual review.

All candidate images and video clips identified by the model were subsequently subjected to careful manual review. During the review process, candidate images were examined in conjunction with adjacent video frames to confirm whether the recorded subject could be reliably identified as a Chinese giant salamander. Particular attention was paid to excluding false positives caused by rocks, branches, water reflections, floating debris, shadows, or other animals. Images confirming the presence of Chinese giant salamanders are shown in Figure 2. Records where the presence of a Chinese giant salamander could not be determined with sufficient confidence were classified as “uncertain” and excluded. Only records that passed both the automated screening and manual confirmation were included in the subsequent detection frequency analysis. Manual review was used solely to confirm the presence of the species and was not used to classify individual behavioral states or quantify behavioral intensity.

Three concepts were distinguished: confirmed detection records, independent detection events, and individual abundance. A confirmed detection record referred to an image or video clip manually verified as containing a Chinese giant salamander. Adjacent records at the same monitoring site were treated as separate independent detection events when the time interval exceeded 30 min; continuous appearances within 30 min were consolidated into one event. Because individual tagging was not conducted and different individuals could not be reliably distinguished in all footage, neither confirmed detection records nor independent detection events represented the number of individual Chinese giant salamanders or were used to estimate population density. Table 1 shows the monitoring and confirmation status of the Chinese giant salamander.

**Figure 3:**
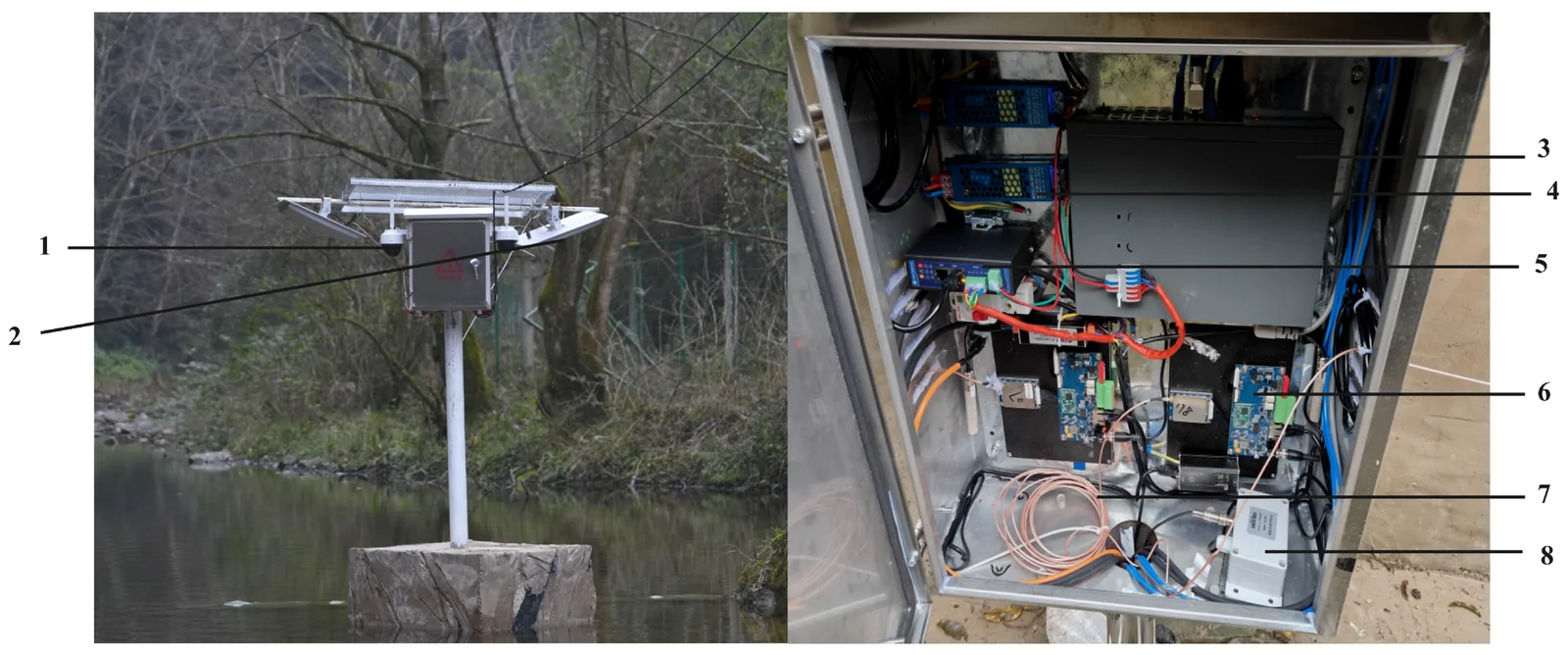
Schematic diagram of image acquisition device. 1. Camera for image capture. 2. Antenna for positioning. 3. Switch device. 4. Power supply unit. 5. Gateway interface. 6. Motherboard for positioning module. 7. Extension cable for positioning antenna. 8. Environmental sensor.

**Figure 4:**
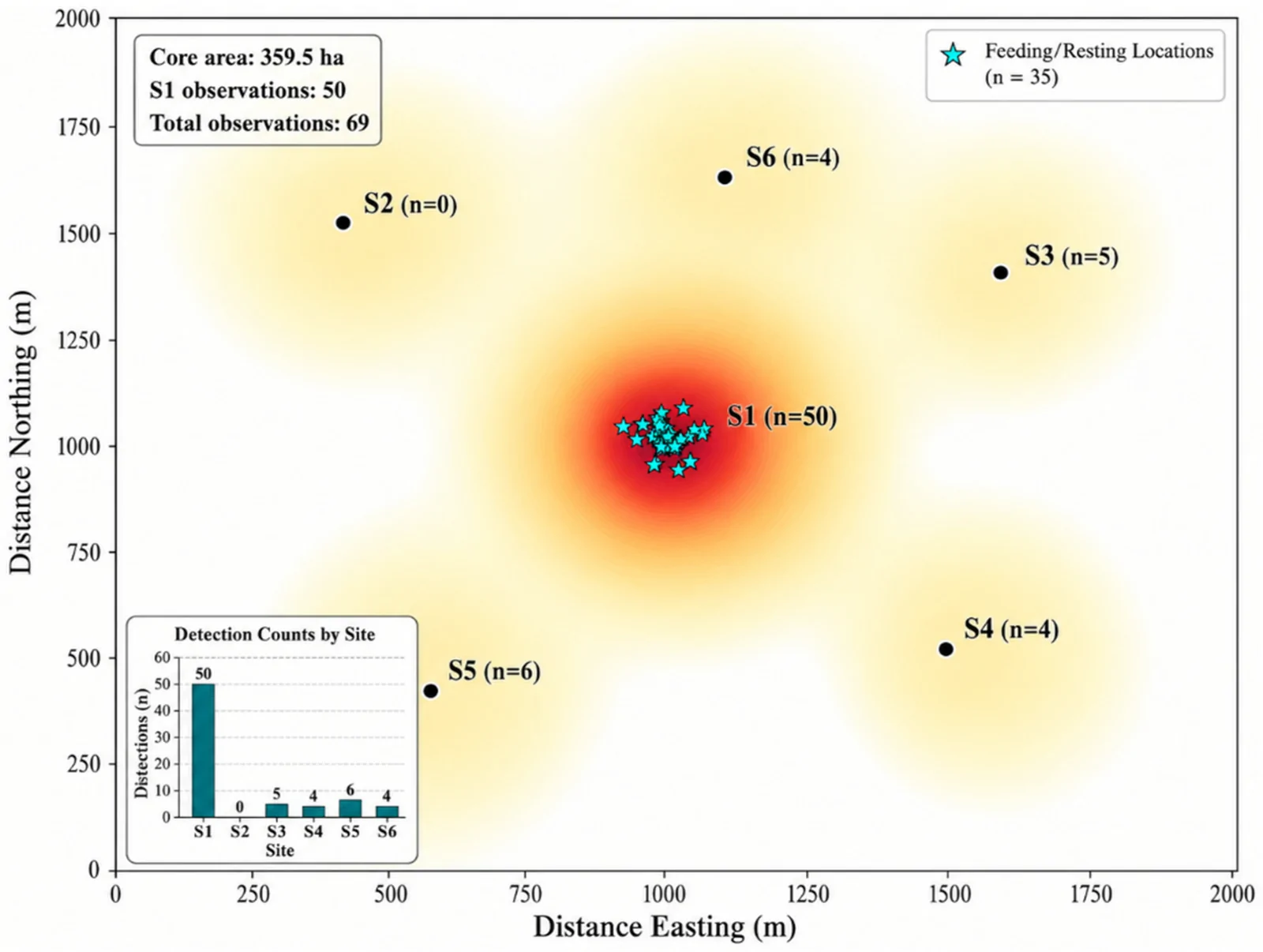
Heat map showing the spatial distribution and frequency of camera-confirmed Chinese giant salamander detection records at the six monitoring sites in the Zhongjian River Giant Salamander National Nature Reserve, Xianfeng, Hubei. Site labels indicate the number of confirmed detection records obtained during the monitoring period, whereas the star symbols at S1 represent 35 independent detection events.

During the monitoring period, S1 had the highest numbers of confirmed detection records and independent detection events and was therefore designated as the high-frequency detection site. Sites S2–S6 were treated as comparison sites for descriptive site-level analyses of stream-water bacterial communities. Monitoring equipment for the Chinese giant salamander is shown in Figure 3. This designation represents relative Chinese giant salamander detection frequency under the present monitoring configuration and period. It does not indicate greater individual abundance, higher behavioural intensity, or core-habitat status. The monitoring frequency and spatial distribution of the Chinese giant salamander are shown in Figure 4.

### 2.3. Sample Collection

Water samples for microbial analysis were collected from six stream sites, S1–S6, within the Zhongjian River Giant Salamander National Nature Reserve. Three spatially adjacent water subsamples, each with a volume of 1–2 L, were collected at each site. To obtain an integrated site-level bacterial community sample, the three adjacent subsamples were pooled in equal volumes before filtration, producing one composite sample per site for filtration, DNA extraction, and 16S rRNA amplicon sequencing. The final dataset therefore comprised six valid sequencing samples corresponding to the six stream sites.

Because the three adjacent subsamples were pooled before sequencing, they could not be treated as independent biological replicates for statistical inference. This study followed an exploratory site-comparison design in which the six stream sites represented the observational units. All bacterial community analyses were used to describe site-level taxonomic composition, diversity, and spatial patterns and were not intended for rigorous inferential comparisons between the high-frequency detection site and the surrounding comparison sites.

Contamination-control procedures were followed throughout sampling. Researchers wore sterile gloves and used disposable sterile sampling containers [34]. A field blank was established at each sampling site to monitor potential external contamination during sample collection and subsequent processing.

Water samples were vacuum-filtered within 4 h of collection using 0.22 μm pore-size membranes, and the filtration volume and membrane retention information were recorded [35]. After filtration, membranes were immediately transferred to sterile cryovials and stored at −80 °C. All samples were transported to the laboratory within 24 h to preserve the original bacterial community information as completely as possible [36,37,38].

### 2.4. Determination of Environmental Factors

The environmental variables were selected to provide a focused characterization of physicochemical conditions relevant to both Chinese giant salamander habitat conditions and stream-water bacterial communities. Water temperature was included because it is a fundamental determinant of amphibian physiological activity and microbial metabolic processes. pH characterizes the acid–base environment and may influence microbial enzyme activity, nutrient availability, and bacterial community assembly. Dissolved oxygen is closely related to aquatic habitat quality and the oxygen requirements of the Chinese giant salamander, while also regulating aerobic and anaerobic microbial processes. Specific conductivity was measured as an indicator of dissolved ionic conditions and potential catchment-derived inputs. Ammonia nitrogen and nitrate nitrogen were selected to characterize inorganic nitrogen status, nutrient loading, and potential nitrogen-related pollution, which may affect both aquatic habitat quality and bacterial community composition. These variables were therefore intended to represent key thermal, acid–base, oxygen, ionic, and nutrient dimensions of the stream environment rather than to provide a complete characterization of all microhabitat factors.

During sampling, a portable multiparameter water quality analyzer (ProPlus; YSI Incorporated, Yellow Springs, OH, USA) was used to measure water temperature (T), pH, dissolved oxygen (DO), and conductivity (SpC) in the surface water (at a depth of approximately 0.3 m) at each sampling site. Three measurements were taken at each site, and the average value was calculated. Additionally, 500 mL of water samples were collected in sterile polyethylene bottles, refrigerated at 4 °C during transport to the laboratory, and analyzed for water quality parameters within 24 h. Ammonia nitrogen (NH_3_-N) was determined using Nessler’s reagent spectrophotometric method in accordance with HJ 535-2009 [39] and nitrate nitrogen (NO_3_^−^-N) was determined using the UV spectrophotometric method in accordance with HJ/T 346-2007 [40]. All environmental parameter measurements were conducted with three technical replicates.

In addition to the synchronous measurements obtained during microbiome sampling, long-term environmental sensor records from S1–S6 were available in the Supplementary Dataset and were used to characterize temporal environmental variability and constancy. These sensor records were analyzed separately from the single-time-point measurements reported in Table 3. For dissolved oxygen, the mean, standard deviation, range, and coefficient of variation were calculated for each site. The coefficient of variation was calculated as the standard deviation divided by the mean and multiplied by 100%, with a lower value indicating lower relative temporal variability. The long-term records were used to describe environmental stability rather than to replace the physicochemical measurements obtained at the time of microbial sampling.

### 2.5. PCR Amplification and 16S rRNA Sequencing

Following the manufacturer’s instructions, total microbial DNA was extracted from the microorganisms retained on the filter membranes of each water sample using an aquatic microbial DNA extraction kit [41]. DNA concentration and purity were measured using a Nanodrop 2000 spectrophotometer (Thermo Scientific, Waltham, MA, USA), and DNA integrity was assessed via 1% agarose gel electrophoresis. The molecular analysis in this study focused on aquatic bacterial communities, rather than species-specific DNA detection of the Chinese giant salamander. For the V3–V4 hypervariable regions of the bacterial 16S rRNA gene, PCR amplification was performed using the barcoded universal primers 341F (5′-CCTAYGGGRBGCASCAG-3′) and 806R (5′-GGACTACNNGGGTATCTAAT-3′).

After detecting the PCR amplification products via 2% agarose gel electrophoresis, the target bands were recovered and quantified. The amplification products from each sample were mixed at equimolar concentrations to construct sequencing libraries, which were then subjected to paired-end sequencing on the Illumina platform by Beijing Novogene Technology Co., Ltd. (Novogene, Beijing, China) [42].

### 2.6. Sequencing Data Processing and Statistical Analysis

Raw paired-end sequencing reads were first quality-filtered using fastp. Sequences containing more than five ambiguous bases or with a proportion of low-quality bases exceeding 15% were removed. Qualified paired-end reads were merged using FLASH v1.2.11 and imported into the QIIME 2 workflow (release 2025.4). Sequence denoising, dereplication, and chimera removal were performed using the DADA2 plugin to generate amplicon sequence variants (ASVs). Taxonomic classification was conducted using the classify-sklearn algorithm against the SILVA 138.2 database. To reduce the effect of differences in sequencing depth, all samples were rarefied to the minimum depth of 69,135 reads. Sequencing quality-control statistics are provided in Supplementary Table S1, the ASV feature table in Supplementary Table S2, and representative ASV sequences and taxonomic annotations in Supplementary Table S3. Following the analytical and presentation framework adopted in recent microbial sequencing research [43], sequencing depth was evaluated using alpha-rarefaction curves based on observed ASVs.

Alpha-diversity indices, including Chao1 richness, observed features, Shannon diversity, Simpson diversity, Pielou’s evenness, dominance, and Good’s coverage, were calculated from the rarefied ASV table. Beta diversity was characterized using Bray–Curtis, Weighted UniFrac, and unweighted UniFrac distances. Principal coordinate analysis (PCoA), non-metric multidimensional scaling (NMDS), and UPGMA clustering were used to visualize site-level bacterial-community dissimilarities. PERMANOVA, ANOSIM, and PERMDISP were considered but not conducted because each site was represented by one composite sequencing sample and the high-frequency detection category contained only S1. Under this design, within-category dispersion could not be estimated, and formal group-level beta-diversity testing would not provide valid statistical inference. The ordination and clustering analyses were therefore interpreted descriptively [44].

Relationships between environmental conditions and bacterial community structure were explored using redundancy analysis (RDA), envfit environmental-vector fitting, Mantel tests, and partial RDA. Community abundance data were Hellinger-transformed, and environmental variables were standardized before analysis. To reduce the risk of overfitting associated with the small sample size, pH, mean dissolved oxygen, and the coefficient of variation in dissolved oxygen were prioritized for inclusion in the constrained ordination model. Mantel tests were used to evaluate the correlation between the Bray–Curtis community-distance matrix and the environmental-distance matrix. A total of 9999 permutations were used for all permutation-based tests.

Variation-partitioning analysis (VPA) was conducted to explore the proportions of bacterial-community variation associated with the measured environmental variables and site category. Site category distinguished S1, the high-frequency detection site, from the S2–S6 comparison sites. Because the high-frequency detection category contained only S1, the VPA results were treated as exploratory descriptions rather than evidence that Chinese giant salamander detection frequency independently explained bacterial-community variation.

Because the final analysis included only six site-level composite samples and lacked independent sequencing replicates, differential-abundance methods such as LEfSe, DESeq2, and ANCOM were not applied. Comparisons at the phylum, class, order, family, and genus levels were based on descriptive relative-abundance patterns and were not interpreted as significant enrichment, statistically validated biomarkers, or functional evidence. Complete RDA, envfit, Mantel, partial RDA, and VPA results are provided in Supplementary Table S4, and the main analytical workflow, software versions, and parameters are provided in Supplementary Table S5.

## 3. Results

### 3.1. Overview of ASVs and Diversity Analysis

The 16S rRNA gene amplification products from all samples were analyzed using paired-end sequencing on the Illumina platform. Following rigorous quality control and chimera filtering, we obtained a total of 5,062,176 high-quality effective tags. Unlike traditional 97% similarity clustering methods, this study identified a total of 40,607 unique sequences (ASVs) using the DADA2 denoising workflow. Detailed statistical results of data processing for each sample are presented in Supplementary Table S1.

### 3.2. Monthly Variation in Confirmed Chinese Giant Salamander Detection Frequency

Between July 2024 and January 2026, a total of 47 independent detection events of the Chinese giant salamander were recorded at the six monitoring sites: 35 at S1, none at S2, 4 at S3, 3 at S4, 2 at S5, and 3 at S6. The distribution of independent detection events by month is shown in Table 2. The monthly counts are used to describe the temporal variation in the frequency of confirmed detections under the current camera monitoring conditions.

### 3.3. Visual Analysis of Microbial Communities

**Figure 5:**
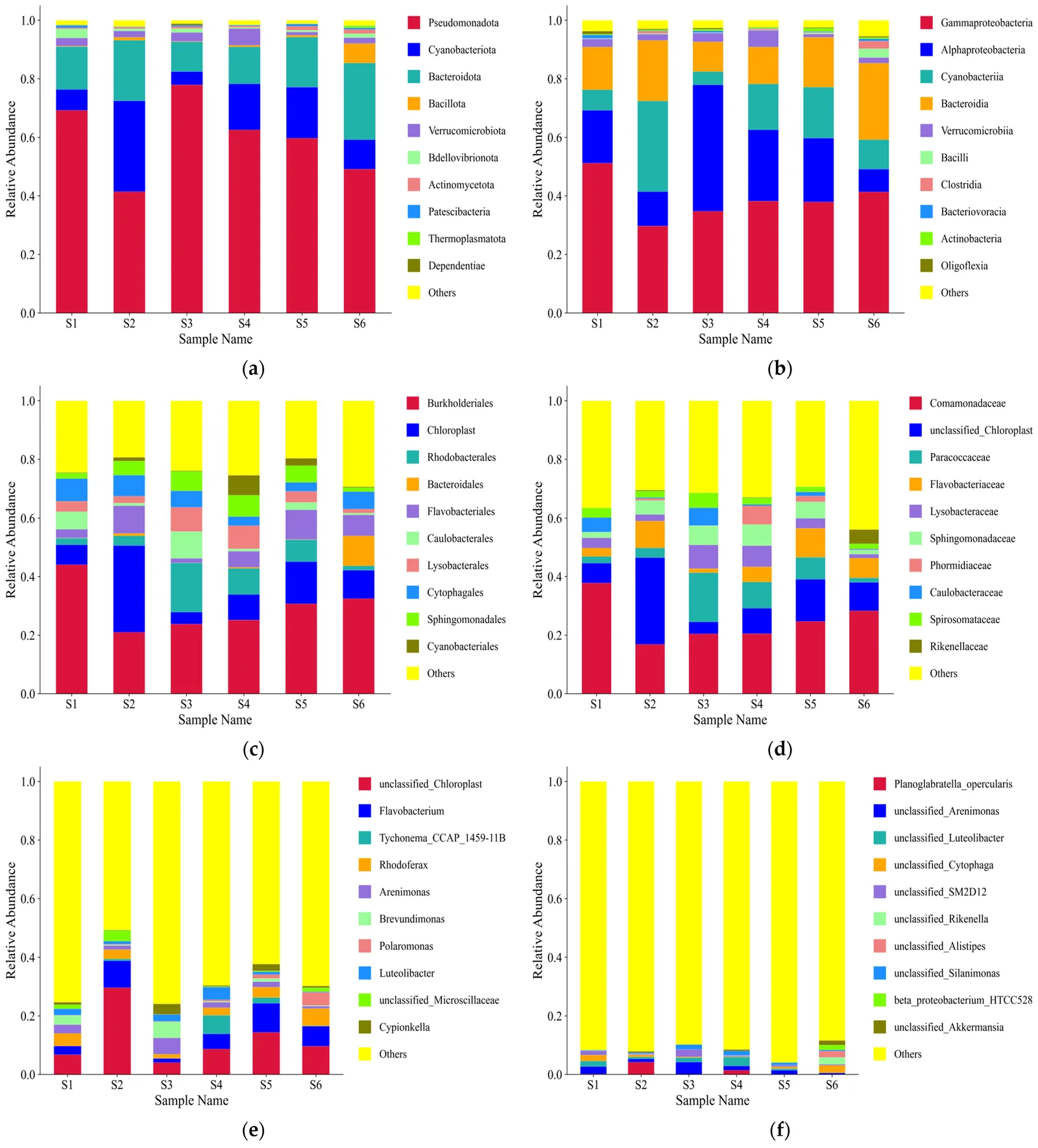
Relative-abundance profiles of the ten most abundant bacterial taxa across the six sampling sites. Panels show the bacterial community composition at the (**a**) phylum, (**b**) class, (**c**) order, (**d**) family, (**e**) genus, and (**f**) species levels. Taxa outside the ten most abundant groups at each taxonomic level were combined as “Others”.

At the phylum level (Figure 5a), Pseudomonadota and Bacteroidota were among the most abundant bacterial phyla across the six sampling sites. Pseudomonadota accounted for more than 65% of the bacterial community at S1. Cyanobacteriota represented approximately 30% of the community at S2, a higher proportion than that observed at the other sites, whereas Bacillota showed a relatively high proportion at S6. These results represent relative-abundance patterns in the site-level composite samples and do not indicate statistically significant differences among sites.

At the class level (Figure 5b), Gammaproteobacteria showed relatively high abundances at S1, S4, S5, and S6. Cyanobacteriia accounted for a relatively large proportion at S2, whereas Alphaproteobacteria were comparatively abundant at S3. Bacteroidia were detected across all six sites.

At the order level (Figure 5c), Burkholderiales accounted for relatively large proportions at S1, S5, and S6, with the highest proportion observed at S1. Chloroplast-related sequences were prominent at S2, Rhodobacterales were relatively abundant at S3, and Flavobacteriales were primarily represented at S1, S4, and S5.

At the family level (Figure 5d), Comamonadaceae showed a relatively high abundance at S1. S2 was characterized by a high proportion of unclassified_Chloroplast sequences, Paracoccaceae were relatively abundant at S3, and Flavobacteriaceae accounted for a comparatively high proportion at S5. Sphingomonadaceae were also represented at S1 and S5.

At the genus level (Figure 5e), *Rhodoferax* and *Flavobacterium* showed relatively high abundances at S1. S2 was dominated by unclassified_Chloroplast sequences, *Arenimonas* was comparatively abundant at S3, and *Brevundimonas* was represented at S1 and S5. *Tychonema* CCAP 1459-11B was mainly detected at S2, S4, and S5.

At the species level (Figure 5f), a substantial proportion of sequences could not be resolved among the ten most abundant classified taxa and were assigned to “Others”. Unclassified *Arenimonas* accounted for a relatively large proportion at S3. Overall, the six site-level composite samples displayed taxonomic heterogeneity; however, these patterns should be interpreted descriptively and do not constitute evidence of significant enrichment or stable microbial biomarkers.

### 3.4. Characteristics of Environmental Factors at Each Sampling Site

The physicochemical parameters measured at the six sampling sites are presented in Table 3. During microbiome sampling, S1 had a dissolved-oxygen concentration of 8.52 mg L^−1^, an ammonia-nitrogen concentration of 0.08 mg L^−1^, and a slightly alkaline pH of 7.82. S2 had comparatively high ammonia-nitrogen and conductivity values and a lower dissolved-oxygen concentration. These measurements indicate site-level differences in water conditions during sampling. They did not independently explain bacterial-community variation or demonstrate that the bacterial characteristics observed at S1 were caused by Chinese giant salamander detection frequency or by specific bacterial groups.

**Table 3:** Environmental parameters at each sampling site (mean ± standard deviation, *n* = 3).

Site	Water Temperature (°C)	pH	DO (mg/L)	NH_3_-N (mg/L)	NO_3_^−^-N (mg/L)	Conductivity (μS/cm)
S1	16.2 ± 0.3	7.82 ± 0.11	8.52 ± 0.18	0.08 ± 0.02	0.42 ± 0.05	185 ± 12
S2	17.5 ± 0.4	8.16 ± 0.14	7.22 ± 0.24	0.35 ± 0.06	1.23 ± 0.11	312 ± 18
S3	16.8 ± 0.2	7.95 ± 0.09	8.01 ± 0.15	0.14 ± 0.03	0.67 ± 0.08	208 ± 14
S4	16.5 ± 0.3	7.88 ± 0.10	8.23 ± 0.20	0.11 ± 0.02	0.58 ± 0.06	195 ± 11
S5	16.3 ± 0.2	7.79 ± 0.08	8.35 ± 0.17	0.09 ± 0.01	0.49 ± 0.04	189 ± 10
S6	16.9 ± 0.3	7.92 ± 0.12	8.12 ± 0.19	0.12 ± 0.03	0.62 ± 0.07	201 ± 13

### 3.5. Alpha Diversity Analysis

**Figure 6:**
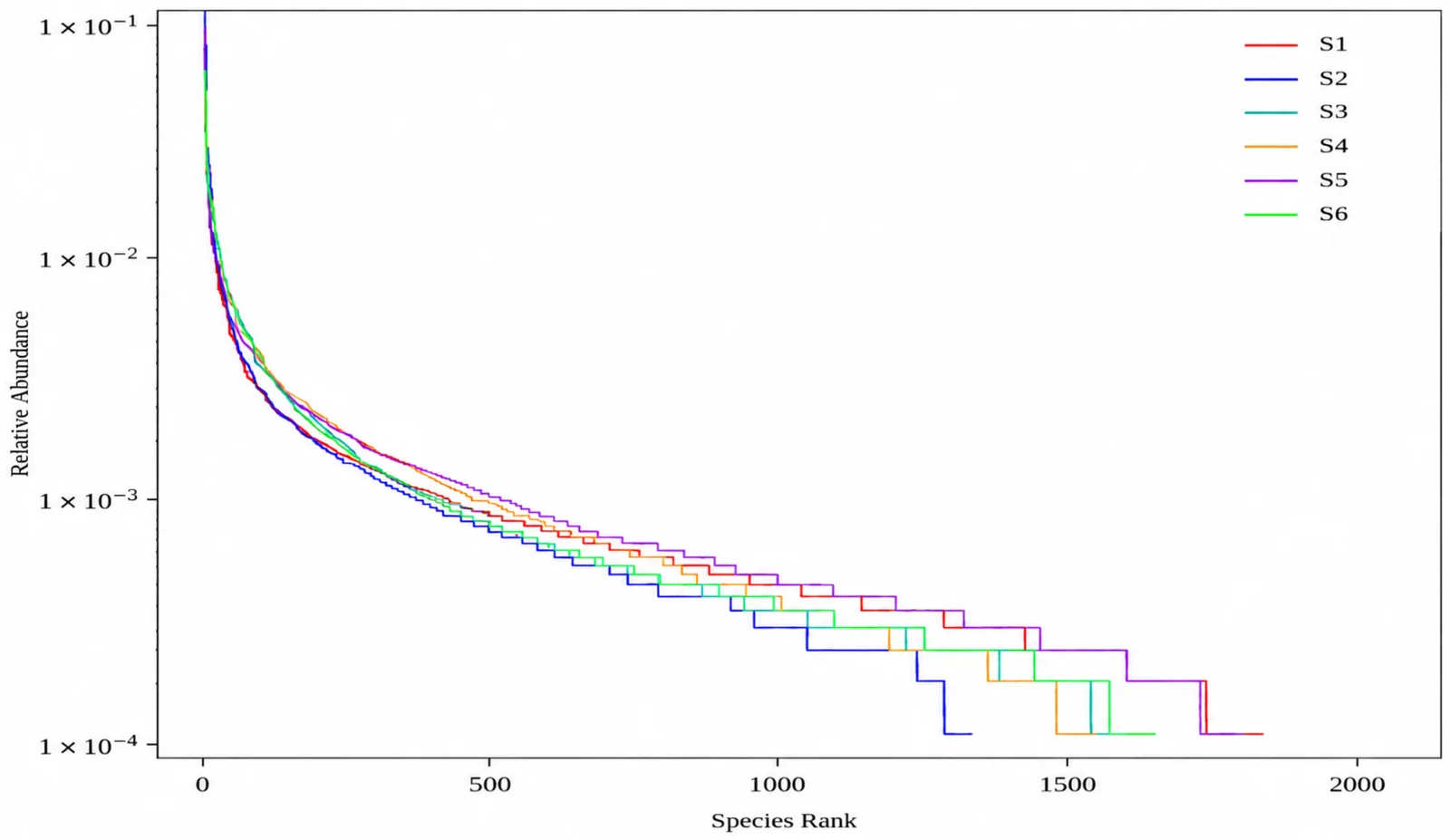
Rank-abundance curves of stream-water bacterial communities across sites S1–S6. The *x*-axis represents ASVs ranked by relative abundance, and the *y*-axis represents relative abundance on a logarithmic scale.

Site-level alpha-diversity estimates are presented in Table 4. S1 had Chao1 and observed-feature estimates of 1833.235 and 1799, respectively, placing it among the sites with comparatively high estimated richness. S4 and S5 showed the highest Shannon-diversity and Pielou’s-evenness values, whereas S2 had lower richness and diversity estimates. Good’s coverage was 0.999 at all six sites, consistent with the sequencing completeness indicated by the rarefaction curves. See Figure 6.

Each site was represented by one composite sequencing sample. The alpha-diversity indices therefore describe site-level patterns and relative ordering among samples rather than statistically tested differences between detection categories.

### 3.6. Beta Diversity Analysis

**Figure 7:**
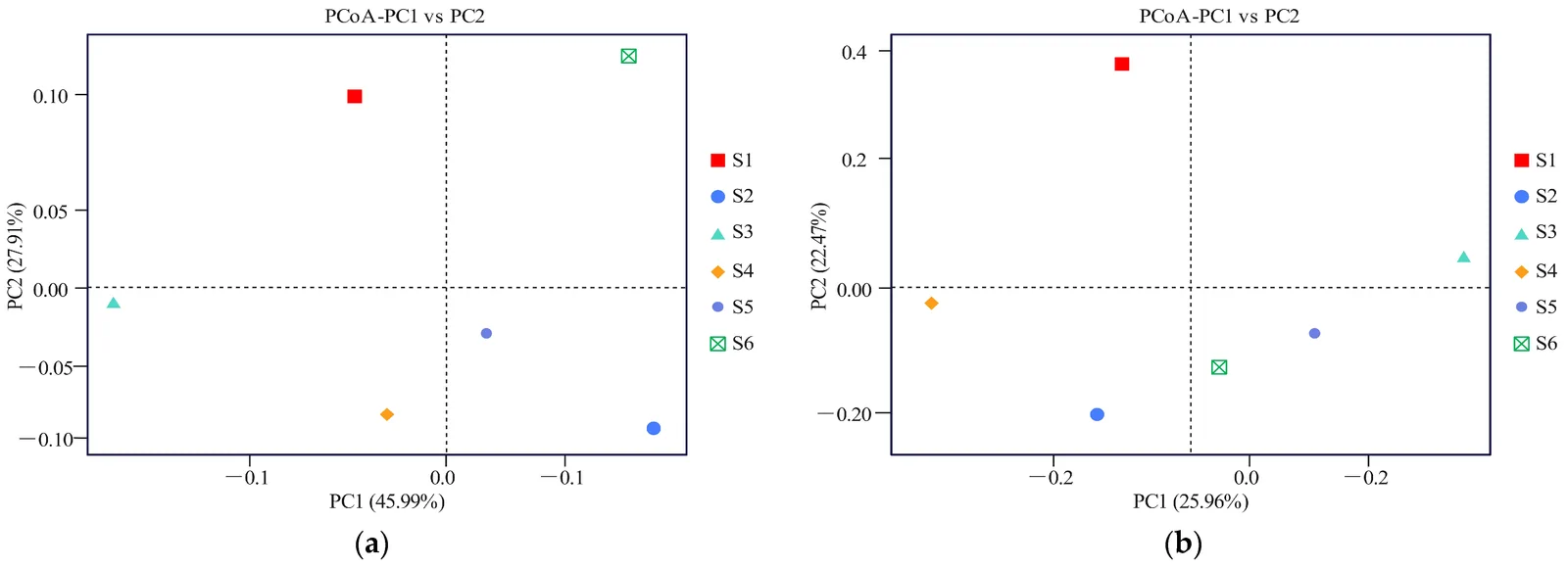
Two-dimensional PCoA plots ((**a**): based on Weighted UniFrac distance; (**b**): based on uweighted UniFrac distance).

**Figure 8:**
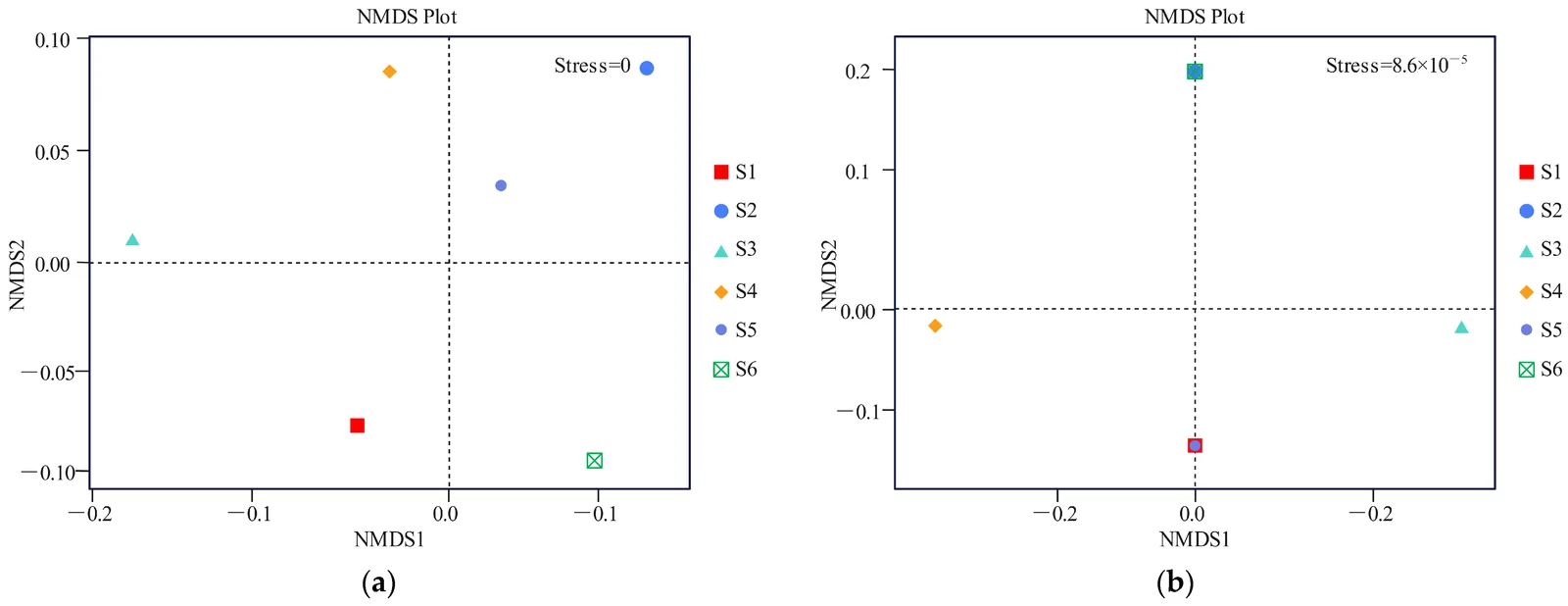
NMDS plots ((**a**): based on Weighted Unifrac distance; (**b**): based on unweighted UniFrac distance).

**Figure 9:**
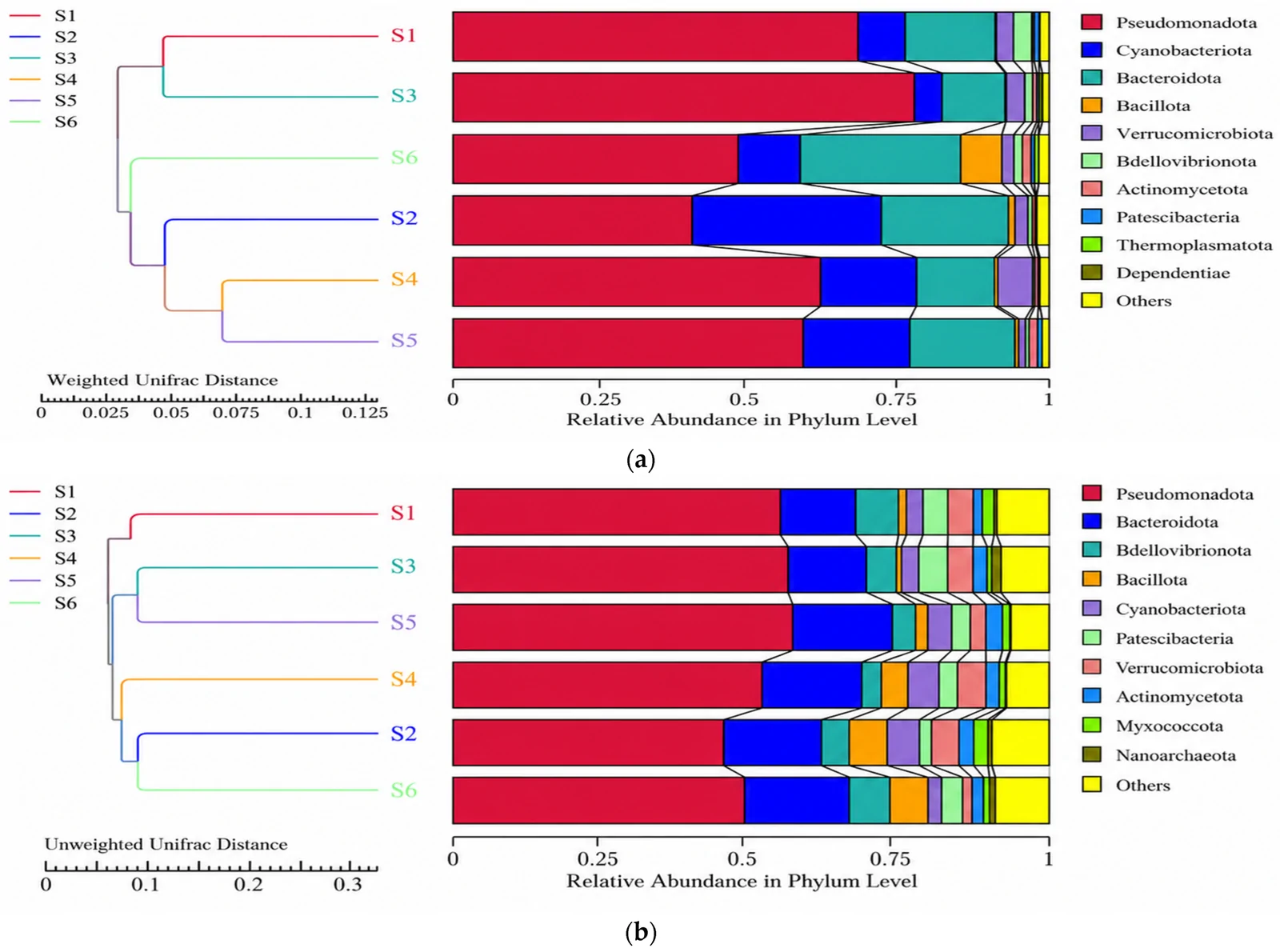
UPGMA clustering tree. ((**a**): based on Weighted Unifrac; (**b**): based on unweighted Unifrac).

PcoA, NMDS, and UPGMA clustering based on weighted and unweighted UniFrac distances revealed spatial heterogeneity in stream-water bacterial-community composition among the six sampling sites (Figure 7, Figure 8 and Figure 9). S1 was separated from several comparison sites in the ordination space, whereas S4 and S5 were positioned relatively close to each other. Weighted UniFrac patterns reflected differences in both phylogenetic composition and relative abundance, while unweighted UniFrac patterns emphasized differences in taxon presence and absence.

The proportions of variation represented by the first two PCoA axes are shown on the axis labels in Figure 7, and the NMDS stress values are reported in Figure 8. These indicators were used to evaluate the representation quality of the ordination results.

The ordination and clustering patterns are descriptive. Each site was represented by one composite sequencing sample, and the high-frequency detection category contained only S1. PERMANOVA, ANOSIM, and PERMDISP were therefore not conducted because valid within-category dispersion could not be estimated. The observed separation represents site-level community dissimilarity rather than statistically confirmed differentiation between high- and low-frequency detection categories.

### 3.7. Analysis of the Association Between Environmental Factors and Bacterial Community Structure

RDA, envfit, Mantel, partial RDA, and VPA were conducted to evaluate associations between the measured environmental variables and aquatic bacterial community structure. RDA1 and RDA2 explained 11.5% and 7.1% of the community variation, respectively. The overall RDA model was not statistically significant (F = 0.19, *p* = 0.319), and the adjusted coefficient of determination was negative (R^2^adj = −0.941), indicating weak explanatory capacity of the included environmental variables under the current sample size and model complexity.

The envfit analysis indicated a marginal association between pH and community ordination structure (r^2^ = 0.108, *p* = 0.061), although this relationship did not reach the *p* < 0.05 significance threshold. Neither mean dissolved oxygen (r^2^ = 0.073, *p* = 0.426) nor DO_CV (r^2^ = 0.082, *p* = 0.264) showed a significant association with community ordination. The Mantel test also failed to identify a significant relationship between Bray–Curtis community dissimilarity and environmental distance (r = 0.221, *p* = 0.337), and the partial RDA was not significant (F = 0.95, *p* = 0.166).

The VPA indicated that 59.8% of bacterial-community variation remained unexplained. The fractions associated with the measured environmental variables and site category were 23.5% and 17.8%, respectively. Because the high-frequency detection category contained only S1, these fractions were interpreted as exploratory descriptions. They do not demonstrate that Chinese giant salamander detection frequency independently explained bacterial-community variation.

**Figure 10:**
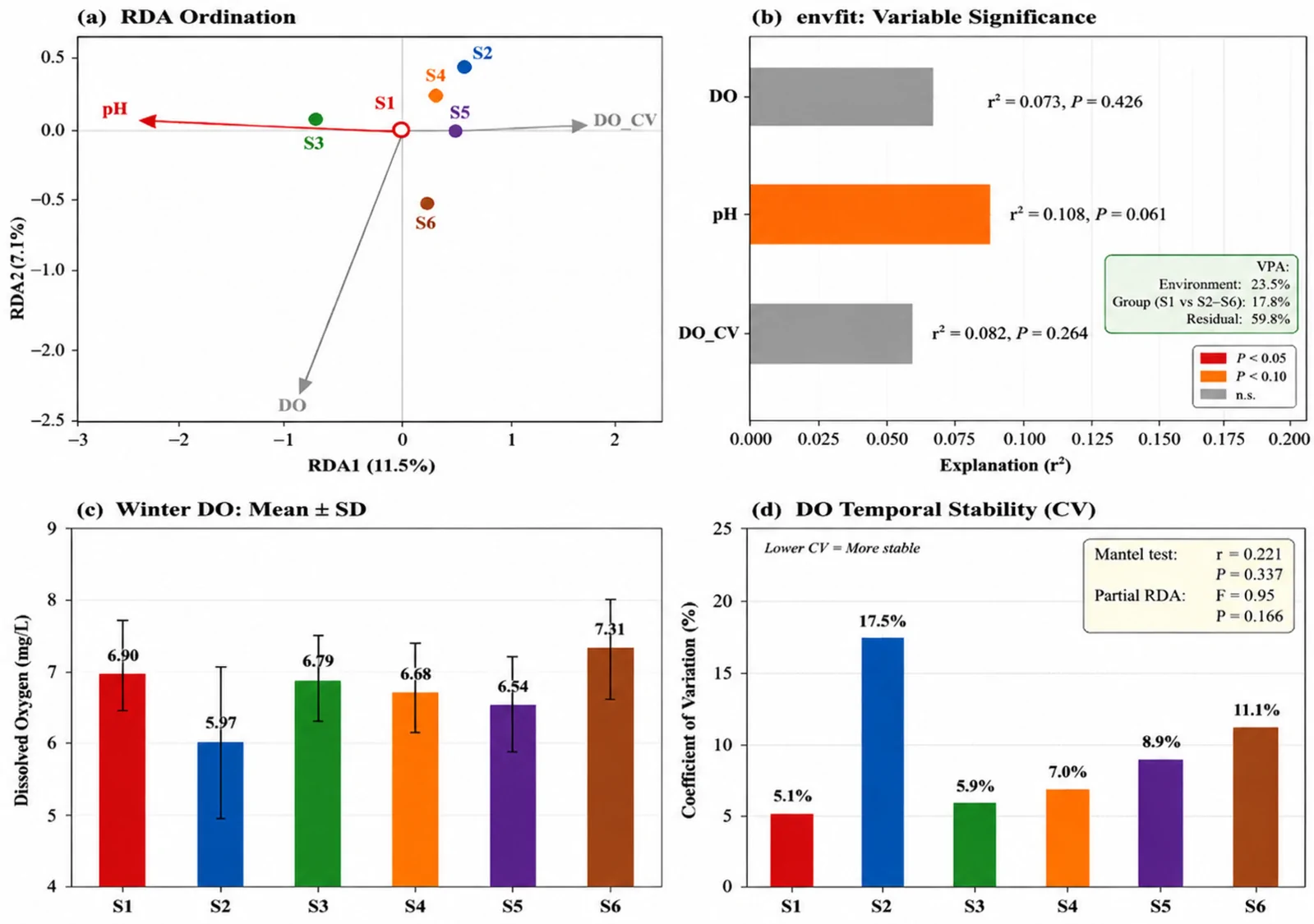
Analysis of the relationship between measured environmental factors and bacterial community structure in water bodies, and analysis of dissolved oxygen stability. (**a**) RDA ordination plot based on pH, DO, and DO_CV; (**b**) envfit results for environmental factor fitting and VPA explanation rate; (**c**) mean and standard deviation of DO at each sampling site during winter; (**d**) temporal stability of DO at each sampling site, expressed as the coefficient of variation (CV). The overall RDA model did not reach statistical significance (F = 0.19, *p* = 0.319), and the Mantel test also failed to show a significant correlation between bacterial community distances and environmental factor distances (r = 0.221, *p* = 0.337). A lower DO_CV value indicates higher temporal stability of dissolved oxygen.

High-frequency winter monitoring showed that S1 did not have the highest mean dissolved oxygen concentration but had the lowest DO_CV, at 5.1%, indicating comparatively low temporal variation in dissolved oxygen. S2 had the highest DO_CV, at 17.5%, indicating greater dissolved oxygen fluctuation. Differences in DO_CV describe variation in aquatic environmental stability but do not demonstrate that dissolved oxygen stability directly drove the bacterial community composition observed at S1. See Figure 10 for detailed data.

Overall, the RDA, envfit, Mantel, and partial RDA analyses did not provide evidence that the measured aquatic environmental variables significantly explained bacterial-community variation. Differences between S1 and the comparison sites may also reflect flow velocity, substrate characteristics, riparian shading, channel structure, organic-carbon inputs, seasonal hydrology, and other unmeasured microhabitat factors. Chinese giant salamander detection records and individual water-quality variables were therefore not interpreted as direct drivers of bacterial-community variation.

### 3.8. Shared and Site-Specific ASV Patterns Among Stream-Water Samples

**Figure 11:**
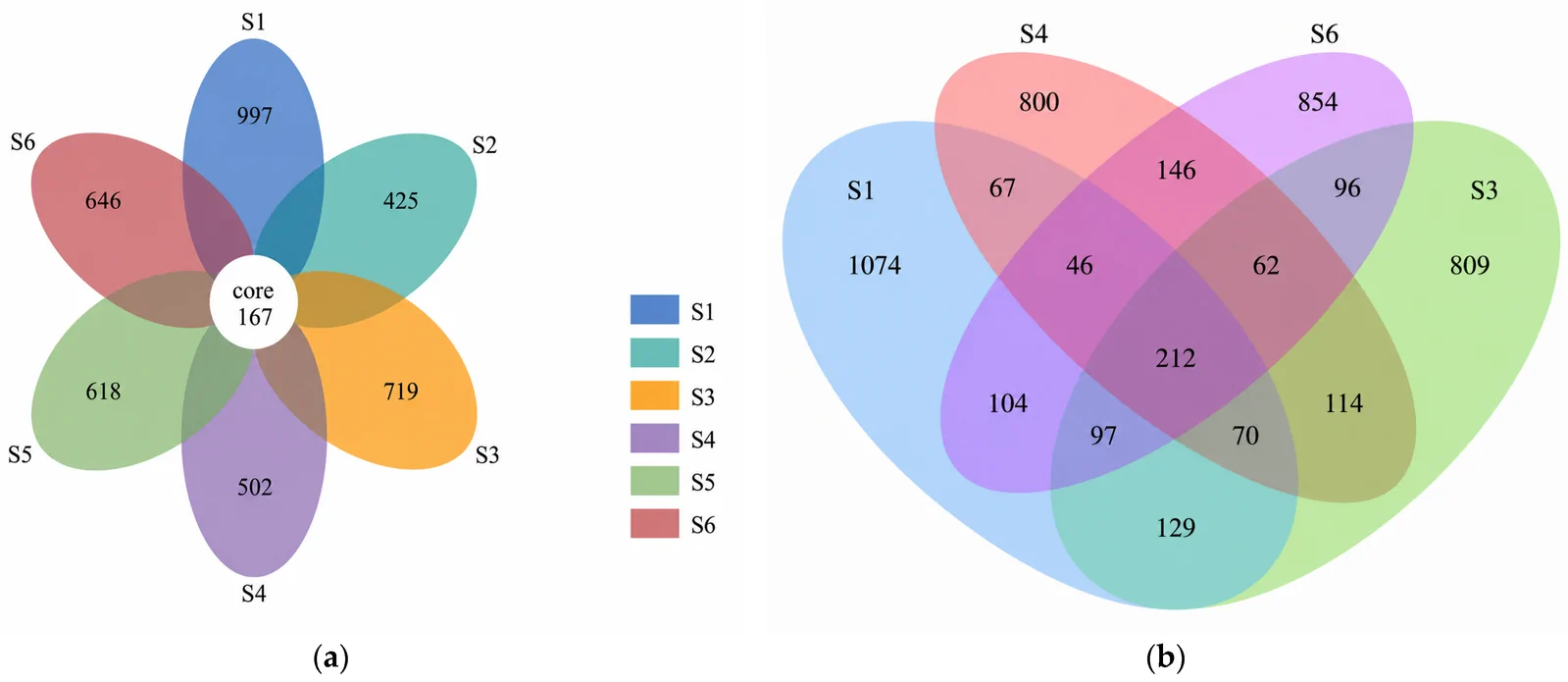
Shared and site-specific ASV patterns among stream-water samples. (**a**) Petal diagram showing the 167 ASVs shared among all six sites and the number of site-specific ASVs at each site, and (**b**) Venn diagram showing shared and site-specific ASVs among S1, S3, S4, and S6.

To compare the shared and unique ASVs across different sampling sites, this study utilized petal plots and Venn diagrams, as shown in Figure 11, to conduct a descriptive analysis of the number of ASVs shared and unique among different sampling sites. The petal plot results showed that the six sampling sites shared 167 core ASVs. In terms of the number of site-specific ASVs, S1 had 997, which was relatively high among the six sampling sites; S2, S4, and S6 had 425, 502, and 646, respectively. Further analysis using Venn diagrams combining S1, S3, S4, and S6 revealed that these four sites shared 212 characteristic sequences, and S1 had 1,074 site-specific ASVs. Since this analysis is based on site-level data and no repeated-measures statistical tests were conducted, this study does not interpret the higher number of ASVs specific to S1 as a difference in the strict statistical sense, nor does it interpret it as evidence of functional advantage or ecological stability. This result merely indicates that S1 exhibits certain distinctiveness in ASV composition and can serve as a candidate lead for subsequent studies involving expanded sample sizes and functional validation.

## 4. Discussion

### 4.1. Spatial Correspondence Between Chinese Giant Salamander Detection Frequency and Stream-Water Bacterial Communities

Integrating long-term computer vision monitoring with 16S rRNA amplicon sequencing revealed spatial correspondence between Chinese giant salamander detection frequency and stream-water bacterial-community characteristics. S1 had the highest numbers of confirmed detection records and independent detection events. It also differed from several comparison sites in taxonomic composition, alpha-diversity estimates, and site-specific ASVs. The two approaches provide complementary habitat information: camera monitoring documents where and when the species was detected, whereas bacterial sequencing characterizes the stream-water microbial environment at the time of sampling.

Computer vision enables long-term wildlife monitoring with limited field disturbance. Detection frequency, however, is affected by camera placement, field of view, illumination, water transparency, target occlusion, equipment operation, animal movement pathways, and model performance [45,46,47,48,49,50,51,52]. The higher detection frequency at S1 therefore reflects the number of confirmed records obtained under the present monitoring configuration. It does not represent individual abundance, population density, locomotor intensity, or core-habitat status.

The bacterial data were obtained from stream-water samples rather than Chinese giant salamander skin, oral, gastric, or intestinal samples. The bacterial characteristics of S1 therefore describe the local aquatic environment. Shared microhabitat conditions may influence both the occurrence of the species and the assembly of stream-water bacterial communities.

### 4.2. Ecological Interpretation of Bacterial Taxa with Relatively High Abundances at S1

Comamonadaceae, *Rhodoferax*, and *Flavobacterium* showed relatively high abundances at S1. These taxa are widely reported in freshwater environments and are often associated with organic-matter transformation, nutrient cycling, and local physicochemical conditions. *Rhodoferax* strains have also been detected in contaminated aquifers and groundwater-treatment systems, where some members participate in the degradation of sulfolane and other organic contaminants [53,54]. The genus *Flavobacterium* includes both environmental and cold-adapted lineages and fish-associated taxa, including recognized pathogenic groups [55,56,57,58,59,60]. In the present study, these taxa represent prominent components of the S1 stream-water bacterial community rather than validated functional or health-related indicators.

Formal differential-abundance analyses, including LEfSe, DESeq2, and ANCOM, were not performed because each site was represented by one composite sequencing sample. The observed taxa should therefore not be described as statistically enriched biomarkers. The 16S rRNA data also do not establish their metabolic functions, pathogenicity, or effects on habitat quality. Their abundance patterns may reflect local water chemistry, hydrology, substrate structure, and organic-matter inputs. They can be retained as candidate taxa for repeated monitoring and functional validation.

### 4.3. Potential Links Among Environmental Conditions, Host-Associated Microbiota, and Chinese Giant Salamander Detection Records

Several processes may contribute to the observed spatial patterns. First, environmental filtering may influence both Chinese giant salamander habitat use and bacterial-community assembly. Water temperature, dissolved oxygen, hydrological stability, rocky substrates, caves and crevices, riparian shading, food resources, and human disturbance can shape the suitability of a stream site. The same factors can also select bacterial taxa adapted to local microhabitats. S1 contained most independent detection events and had comparatively low temporal variability in dissolved oxygen, but the measured environmental variables did not significantly explain its bacterial-community composition.

Second, the presence of Chinese giant salamanders may alter small-scale inputs to the aquatic environment. Residence, feeding, excretion, mucus release, and substrate disturbance can change local organic matter, nutrients, and microbial inputs. These processes were not measured in the present study. The camera records documented occurrence and detection frequency but did not quantify feeding, excretion, mucus production, movement intensity, or substrate disturbance.

Third, environmental microbial exposure may interact with host-associated microbiota. In captive-bred Chinese giant salamander larvae, exposure to non-sterile river sediment altered oral bacterial composition. Firmicutes, particularly Lactobacillaceae, decreased, whereas Proteobacteria, especially Gammaproteobacteria, increased [12]. The skin, stomach, and intestinal microbiota were more stable, indicating that environmental effects differed among anatomical niches. The gastrointestinal microbiota of Chinese giant salamanders also varies with age and developmental stage [61,62,63,64]. Studies of farmland frogs and high-altitude yaks further show that diet, habitat conditions, and gastrointestinal microbiota can change together [16,17].

Host-associated microorganisms may also contribute to amphibian health. Protective cutaneous bacteria can reduce infection by *Batrachochytrium dendrobatidis* [18], and variation in frog skin bacterial diversity has been associated with susceptibility to this pathogen [19]. Amphibian skin microbiota can change over time while retaining relatively stable predicted pathogen-inhibitory functions [20]. Environmental fluctuations may further modify interactions among hosts, skin bacteria, and fungal pathogens [21].

These studies support a plausible pathway linking environmental microbial exposure, host-associated microbiota, physiology, and environmental adaptation. The present dataset does not directly test that pathway because it contains stream-water bacterial profiles and site-level detection records rather than host microbiomes, physiological measurements, or individual behavioural trajectories. The current results are best understood as spatial covariation among Chinese giant salamander detections, local environmental conditions, and stream-water bacterial communities.

### 4.4. Environmental Explanatory Capacity and Unmeasured Microhabitat Factors

Stream-water bacterial communities respond to multiple environmental processes. In addition to water temperature, pH, dissolved oxygen, conductivity, and nutrients, community composition may be influenced by flow velocity, substrate type, riparian shading, stream width, organic-carbon inputs, channel microtopography, and seasonal hydrology.

The overall RDA model was not significant, and the adjusted coefficient of determination was negative. The envfit analysis identified a marginal association between pH and community ordination, but the relationship did not reach the significance threshold. Mean dissolved oxygen and DO_CV were not significant. The Mantel test and partial RDA also found no significant associations. Variation partitioning left 59.8% of bacterial-community variation unexplained.

S1 had the lowest DO_CV, indicating relatively low temporal variation in dissolved oxygen, but this pattern did not account for its bacterial-community composition. The differences among sites are more likely to reflect interactions among measured water-quality variables and unmeasured characteristics such as flow, substrate, shading, refuges, organic-matter inputs, food availability, and anthropogenic disturbance. No single environmental factor or bacterial group adequately explained the spatial patterns.

### 4.5. Management Implications for the Nature Reserve

The integration of camera-confirmed Chinese giant salamander detections, physicochemical water-quality measurements, habitat characteristics, and stream-water bacterial-community profiles provides a practical framework for monitoring habitats in the Zhongjian River Nature Reserve. Monitoring should be conducted at three complementary levels. First, continuous camera monitoring should be maintained at S1 and gradually extended to stream sections containing caves, rock crevices, concealed channels, and other potential refuges. Second, seasonal surveys should combine camera records with measurements of water temperature, dissolved oxygen, pH, conductivity, nutrients, flow velocity, turbidity, substrate composition, and riparian shading. Stream-water bacterial communities should be sampled during the same surveys to characterize changes in local microecological conditions. Third, event-triggered monitoring should be conducted after heavy rainfall, flooding, abrupt dissolved-oxygen decline, abnormal turbidity, pollution incidents, or intensified human disturbance.

Habitat management should prioritize stream reaches with low human disturbance, suitable dissolved-oxygen conditions, heterogeneous rocky substrates, intact caves and crevices, continuous riparian vegetation, and hydrological connectivity. Activities that increase sediment deposition, damage riverbank vegetation, block stream connectivity, disturb refuge structures, or introduce pollutants should be strictly controlled. S1 should be retained as a priority monitoring site because it had the highest frequency of confirmed detections during the monitoring period. However, monitoring and management should also cover the surrounding comparison sites and other potentially suitable stream reaches rather than concentrating conservation resources at S1 alone.

Changes in Chinese giant salamander detection frequency should be evaluated together with water-quality, hydrological, habitat-structure, and microbial information. A decline in confirmed detections accompanied by deteriorating dissolved-oxygen conditions, increased nutrient concentrations, habitat disturbance, or marked changes in bacterial-community composition should prompt additional field investigation and intensified monitoring. Comamonadaceae, *Rhodoferax*, and *Flavobacterium* may be retained as candidate taxa for repeated surveillance because they showed relatively high abundances at S1. These taxa should be incorporated into a broader indicator system rather than used alone to assess habitat quality or guide microbial manipulation. Their temporal stability, ecological functions, pathogenic potential, and relationships with Chinese giant salamander health require validation through repeated multi-season and multi-site monitoring.

### 4.6. Limitations of Computer Vision Monitoring and Aquatic Bacterial Community Analysis in Habitat Assessment

This study has several limitations. First, only one high-frequency detection site and five surrounding comparison sites were included. The bacterial characteristics observed at S1 may therefore reflect site-specific spatial conditions and cannot be generalized to all habitats used by wild Chinese giant salamanders. Second, three adjacent water subsamples from each site were pooled before sequencing, producing only one composite sequencing sample per site. The absence of independent biological replicates prevented reliable differential-abundance testing, estimation of within-site variation, and formal group-level statistical inference. Third, 16S rRNA amplicon sequencing describes bacterial taxonomic composition and relative abundance but cannot directly validate microbial metabolic functions, pathogenicity, temporal activity, or effects on Chinese giant salamander health.

Fourth, computer vision records were influenced by camera placement, illumination, water transparency, target occlusion, equipment operation, and animal movement pathways. Confirmed detection records and independent detection events represented site-level detection frequency rather than individual abundance, population density, or behavioural intensity. Although monthly records described temporal variation in confirmed detections, they could not be used to identify individual inactivity, hyperactivity, behavioural adaptation, or recovery because individual Chinese giant salamanders were not tagged or continuously tracked.

Fifth, the environmental microbiome was characterized during a single sampling campaign, and no repeated temporal microbiome samples were collected. Consequently, this study could not evaluate seasonal microbial dynamics, abrupt replacement of dominant bacterial groups, disturbance-related microbial “crisis” periods, community resistance or recovery, or +1 to +7 day lagged relationships between microbial change and Chinese giant salamander detections. Long-term dissolved-oxygen records described the temporal variability of one environmental factor but could not substitute for repeated microbiome observations. In addition, illumination, precipitation, atmospheric pressure, flow velocity, turbidity, substrate conditions, and several other microhabitat variables were not continuously and synchronously recorded at all sites.

Finally, no salamander oral, skin, gastric, intestinal, or fecal microbiomes, physiological indicators, or host-health data were collected. Therefore, the proposed environmental microbiome–host-associated microbiome–physiology–activity pathway could not be directly tested. Future studies should include multiple independent high-frequency detection sites and matched comparison sites, retain independent biological replicates, and conduct repeated multi-season and event-triggered sampling [49]. Continuous hydrological and meteorological measurements should be synchronized with environmental microbiome sampling. Subject to appropriate conservation and ethical permits, non-invasive skin or fecal microbiome sampling, metagenomic or metatranscriptomic sequencing, functional-gene quantification, potential-pathogen screening, host-health indicators, and individual-level tracking should also be integrated [50].

## 5. Conclusions

This study presents an exploratory spatial-baseline assessment integrating long-term computer vision monitoring, stream-water bacterial-community analysis, synchronous physicochemical measurements, and long-term dissolved-oxygen records in habitats used by the Chinese giant salamander, *Andrias davidianus*. S1 had the highest frequency of camera-confirmed Chinese giant salamander detections and comparatively low temporal variability in dissolved oxygen. Its stream-water bacterial community also differed from several comparison sites in taxonomic composition, site-level alpha-diversity estimates, and site-specific ASVs. Comamonadaceae, *Rhodoferax*, and *Flavobacterium* showed relatively high abundances at S1 and may be retained as candidate taxa for subsequent habitat-monitoring studies.

The environmental, microbial, and Chinese giant salamander detection data provided complementary information at different temporal and spatial scales. Long-term dissolved-oxygen records characterized temporal environmental variation, monthly camera records described changes in confirmed detection frequency, and 16S rRNA sequencing revealed spatial heterogeneity in stream-water bacterial communities. RDA, envfit, Mantel, partial RDA, and variation-partitioning analyses did not identify a significant independent relationship between the measured water-quality variables and bacterial-community variation. The observed spatial correspondence among confirmed detections, environmental conditions, and bacterial-community characteristics probably reflects interactions among water quality, hydrology, substrate structure, riparian shading, refuge availability, organic-matter inputs, food resources, and anthropogenic disturbance.

A key unresolved question is whether changes in the environmental microbiome are followed by delayed responses in Chinese giant salamanders. The present study could not quantify such a time lag because the stream-water bacterial community was characterized during a single sampling campaign and was not repeatedly and synchronously assessed alongside individual-level behavioural observations, physiological indicators, or host-associated microbiomes. Future studies should therefore combine high-frequency, repeated sampling of environmental and host-associated microbiomes with continuous environmental monitoring, physiological measurements, and individual-level tracking. Predefined lagged analyses, for example, over intervals of 1–7 days, could then be used to determine whether microbial changes precede detectable organismal responses and to estimate the duration of any such delay.

For the conservation of *A. davidianus*, environmental microbiome information should be incorporated as a complementary component of habitat assessment together with camera-confirmed detections, physicochemical water quality, hydrological conditions, substrate characteristics, caves and rock crevices, riparian vegetation, meteorological disturbance, and human activity. Management should prioritize the protection of low-disturbance stream reaches, suitable dissolved-oxygen conditions, heterogeneous rocky substrates, natural refuges, intact riparian shading, and hydrological connectivity. Routine seasonal monitoring should be combined with targeted surveys following heavy precipitation, flooding, abrupt water-quality changes, pollution incidents, or intensified human disturbance. Comamonadaceae, *Rhodoferax*, and *Flavobacterium* may be retained as candidate taxa for further validation, but habitat-quality assessment and conservation decisions should be based on integrated ecological evidence rather than on individual microbial groups alone.

## Figures and Tables

**Table 1:** Spatial relationship, habitat characteristics, monitoring effort, and camera-confirmed Chinese giant salamander detections at sites S1–S6.

Site	Distance from S1 (m)	Habitat Description	Monitoring Effort	Confirmed Detection Records	Independent Detection Events	Detection Category
S1	0	Main stream channel, near cave/crevice hiding spots	4 cameras, continuous monitoring	50	35	High-frequency detection site
S2	1600	Areas of slow-moving water or open water	4 cameras, continuous monitoring	0	0	No confirmed detections
S3	1300	Near a tributary with a rocky riverbed	4 cameras, continuous monitoring	5	4	Low-frequency detection site
S4	960	River sections with alternating fast and slow currents	4 cameras, continuous monitoring	4	3	Low-frequency detection site
S5	830	Rock crevices near the shore or sheltered sections of the river	4 cameras, continuous monitoring	6	2	Low-frequency detection site
S6	730	Downstream/Peripheral Comparison River Sections	4 cameras, continuous monitoring	4	3	Low-frequency detection site

Note: The distance to S1 refers to the approximate straight-line distance from each sampling point to S1 and is used solely to characterize the spatial relationship between the surrounding comparison sites and S1. The number of confirmed detection records refers to the number of image frames or video clips identified by the YOLOv11-EWL model and manually verified to contain Chinese giant salamander targets. The number of independent detection events is calculated by treating each interval of more than 30 min between adjacent detection records at the same location as a new, independent event; if the same Chinese giant salamander, or one suspected to be the same individual, appears continuously within the same camera frame for 30 min, these occurrences are combined into a single independent detection event. Neither the number of confirmed detection records nor the number of independent detection events represents the number of individual Chinese giant salamanders, nor are they used to estimate population density.

**Table 2:** Monthly distribution of independent Chinese giant salamander detection events at the six monitoring sites from July 2024 to January 2026.

Month	S1	S2	S3	S4	S5	S6	Monthly Total
Jul. 2024	2	0	0	0	0	0	2
Aug. 2024	3	0	0	0	0	0	3
Sep. 2024	3	0	1	0	0	0	4
Oct. 2024	2	0	0	1	0	0	3
Nov. 2024	1	0	0	0	1	0	2
Dec. 2024	0	0	0	0	0	0	0
Jan. 2025	0	0	0	0	0	0	0
Feb. 2025	1	0	0	0	0	0	1
Mar. 2025	2	0	0	0	0	1	3
Apr. 2025	3	0	1	0	0	0	4
May 2025	3	0	0	1	0	0	4
Jun. 2025	3	0	0	0	1	0	4
Jul. 2025	4	0	1	0	0	1	6
Aug. 2025	3	0	0	1	0	0	4
Sep. 2025	2	0	1	0	0	0	3
Oct. 2025	1	0	0	0	0	1	2
Nov. 2025	1	0	0	0	0	0	1
Dec. 2025	1	0	0	0	0	0	1
Jan. 2026	0	0	0	0	0	0	0
Total	35	0	4	3	2	3	47

Note: Values represent the number of independent detection events rather than the total number of image frames or video clips. Confirmed records occurring at the same monitoring site within 30 min were consolidated into one independent detection event. A value of zero indicates that no camera-confirmed detection event was obtained during that month and does not necessarily indicate biological inactivity.

**Table 4:** Alpha-diversity indices of stream-water bacterial communities at sites S1–S6.

Site	Chao1	Dominance	Good’s Coverage	Observed Features	Pielou’s Evenness	Shannon	Simpson
S1	1833.235	0.025	0.999	1799	0.717	7.750	0.975
S2	1323.204	0.041	0.999	1303	0.691	7.153	0.959
S3	1610.153	0.010	0.999	1589	0.762	8.106	0.990
S4	1537.051	0.010	0.999	1517	0.788	8.322	0.990
S5	1785.693	0.021	0.999	1765	0.772	8.325	0.979
S6	1640.279	0.014	0.999	1617	0.753	8.031	0.986

## Data Availability

Supplementary data for this study have been made publicly available at https://www.mdpi.com/article/10.3390/biology15181647/s1. The remaining data are available upon request from the authors for educational and non-commercial purposes.

## Supplementary Materials

The following supporting information can be downloaded at: https://www.mdpi.com/article/10.3390/biology15181647/s1, Figure S1: Genus-level phylogenetic tree; Table S1: Sequencing quality-control statistics; Table S2: ASV feature table; Table S3: ASV taxonomic assignments; Table S4: RDA envfit Mantel Partial RDA Variation-Partitioning Resu; Table S5: Analytical Workflow Software Versions Parameters; Data S1–S6: Raw winter environmental sensor records from sites S1–S6. File S1: Microbial Data.

## Abbreviations

The following abbreviations are used in this manuscript:

ANCOM	Analysis of Compositions of Microbiomes
ANOSIM	Analysis of Similarities
ASV	Amplicon Sequence Variant
CV	Coefficient of Variation
DADA2	Divisive Amplicon Denoising Algorithm 2
DNA	Deoxyribonucleic Acid
DO	Dissolved Oxygen
DO_CV	Coefficient of Variation of Dissolved Oxygen
LEfSe	Linear Discriminant Analysis Effect Size
NH_3_-N	Ammonia Nitrogen
NMDS	Non-Metric Multidimensional Scaling
NO_3_^−^-N	Nitrate Nitrogen
PCoA	Principal Coordinate Analysis
PCR	Polymerase Chain Reaction
PERMANOVA	Permutational Multivariate Analysis of Variance
PERMDISP	Permutational Analysis of Multivariate Dispersions
QIIME 2	Quantitative Insights Into Microbial Ecology 2
RDA	Redundancy Analysis
rRNA	Ribosomal Ribonucleic Acid
SpC	Specific Conductivity
UPGMA	Unweighted Pair Group Method with Arithmetic Mean
UV	Ultraviolet
VPA	Variation-Partitioning Analysis
YOLO	You Only Look Once
